# Cross‐population validation of the PreMO risk indicator for predicting myopia onset in children

**DOI:** 10.1111/opo.13416

**Published:** 2024-11-18

**Authors:** Jane M. Fulton, Tsz Wing Leung, Sara J. McCullough, Kathryn J. Saunders, Nicola S. Logan, Carly S. Y. Lam, Lesley Doyle

**Affiliations:** ^1^ Centre for Optometry and Vision Science Ulster University Coleraine UK; ^2^ Research Centre for SHARP Vision (RCSV) The Hong Kong Polytechnic University Kowloon Hong Kong; ^3^ School of Optometry, Centre for Myopia Research The Hong Kong Polytechnic University Kowloon Hong Kong; ^4^ Centre for Eye and Vision Research The Hong Kong Polytechnic University, Hong Kong and University of Waterloo Waterloo Ontario Canada; ^5^ School of Optometry Aston University Birmingham UK

**Keywords:** myopia, myopia onset, myopia prediction, PreMO risk indicator, pre‐myopia, risk of myopia

## Abstract

**Purpose:**

The Predicting Myopia Onset and progression (PreMO) risk indicator, developed using data generated from white children in the UK, incorporates age, spherical equivalent refraction (SER), axial length (AL) and parental myopia to stratify the likelihood of developing myopia. This study evaluated the PreMO's predictive accuracy using prospective datasets from independent samples of children in Hong Kong (HK) and an ethnically diverse cohort of children in the United Kingdom.

**Methods:**

Non‐myopic children (SER > –0.50 D) aged 6–8 and 9–10 years were scored using the PreMO risk indicator framework, integrating baseline cycloplegic SER, AL and parental myopia data. Scores were assigned risk categories as follows: 0 = no risk, 1–3 = low risk, 4–6 = moderate risk and 7–9 = high risk. SER at ≥15 years of age was used to define refractive outcomes as ‘myopic’ or ‘not myopic’.

PreMO's predictive accuracy was analysed via Receiver Operator Characteristic curves, with Youden's *J*‐Index identifying the optimal risk score threshold. Sensitivity, specificity and area under the curve were determined and compared with those of singular predictors, that is, SER < +0.75 D and AL ≥ 23.07 mm at 6–8 years.

**Results:**

In the cohort of children aged 6–8 years, a PreMO risk score ≥ 4 exhibited high sensitivity in predicting myopia onset in UK (0.97) and HK (0.94) children, with high specificity in UK (0.96) and moderate specificity in HK (0.64) children. In UK children aged 6–8 years, the PreMO outperformed singular predictors such as SER and AL. Among HK children aged 9–10 years, the PreMO score maintained high sensitivity (0.90) and moderate specificity (0.72).

**Conclusions:**

A PreMO risk score ≥ 4 is a strong predictive indicator for future myopia onset, particularly in UK children. Despite high sensitivity in both UK and HK cohorts, specificity varied, indicating the need for contextual application of the tool, particularly in pre‐myopic Asian children.

**Key points**

- The Predicting Myopia Onset and progression risk indicator is derived from a population‐based study of white children and utilises age, spherical equivalent refraction, axial length and parental myopia to stratify future risk of myopia.
- Demonstrating high sensitivity and specificity, a Predicting Myopia Onset and progression indicator risk score ≥ 4 is strongly indicative of future myopia in children living in the United Kingdom.
- When applied to children living in Hong Kong, a Predicting Myopia Onset and progression indicator risk score of ≥4 was highly sensitive but only moderately specific in its ability to signal children at risk for future myopia.

## INTRODUCTION

The World Health Organisation has identified the escalating prevalence of myopia as a major public health challenge.^1^ This ocular condition extends beyond the mere necessity for optical correction; more concerningly, it markedly increases the risk of multiple sight‐threatening pathologies.^2^, ^3^, ^4^ Contrary to the common belief that only high myopia (≤−6 D) amplifies the risk of ocular diseases, recent research indicates that even low levels of myopia (≤−3 D) can double the risk of myopic maculopathy and posterior subcapsular cataract and triple the risk of retinal detachment compared to the emmetropic eye.^5^

In response to the global epidemic of childhood myopia, leading optometric and ophthalmologic professional organisations around the world have issued resolutions, statements or consensus documents. These bodies unanimously agree that no level of myopia can be considered entirely safe, and assert that current evidence sufficiently justifies the initiation of myopia prevention and control strategies for children at risk of progressive myopia.^6^, ^7^, ^8^, ^9^ The World Council of Optometry has outlined a standard of care for myopia management, stating that ‘simply correcting the refractive error is no longer sufficient, and myopia management should not be optional and rather be an obligation of optometrists’.^6^ This standard of care also emphasises that evidence‐based myopia management should encompass three principal components: mitigation of risk factors, thorough measurement of ocular status and strategic management of myopia.

In standard optometric practice, mitigating the risk of myopia in children at risk of developing myopia—known as ‘pre‐myopes’—typically involves providing prophylactic lifestyle advice to delay the age of onset.^10^, ^11^, ^12^, ^13^, ^14^, ^15^ The International Myopia Institute defines pre‐myopia as ‘a refractive state of an eye close to emmetropia in children where a combination of baseline refraction, age and other quantifiable risk factors provide a sufficient likelihood of the future development of myopia to merit preventative interventions’.^16^ While lifestyle advice serves as a cost‐effective and low‐risk approach, emerging research has highlighted additional preventative measures such as repeated low‐level red‐light therapy and low‐dose atropine. These interventions, though more invasive, have shown efficacy in slowing myopia development.^17^, ^18^, ^19^, ^20^, ^21^

The rate of myopia progression is significantly influenced by the age at which it begins, with each one‐year delay in onset potentially lowering the eventual severity of myopia by up to one dioptre.^22^ Additionally, postponing myopia onset by just 1 year can reduce the risk of developing myopic maculopathy by 40%,^23^ an advantage comparable to the cumulative benefits of several years of myopia control treatment. These substantial health benefits underscore the urgent need for precise myopia prediction models to effectively identify children who are at risk. Conversely, effective myopia prediction models also serve to prevent unnecessary intervention in children with a low likelihood of developing myopia. The development and implementation of such models are particularly crucial for East and Southeast Asian children, whose myopia usually progresses more rapidly than in other ethnic groups.^24^ Consequently, delaying the age of myopia onset is expected to have the largest impact on children of these ethnicities.^22^

Previous research suggests that singular predictors, such as baseline refraction or ocular biometric data, may sufficiently forecast future myopia development.^25^, ^26^, ^27^, ^28^, ^29^ On the other hand, other studies have demonstrated that prediction models integrating these measures with lifestyle or genetic information can yield improved predictive accuracy.^30^, ^31^, ^32^, ^33^ However, more complex models, which include numerous variables, are at risk of overfitting. This overfitting can reduce the models' generalisability and weaken their predictive power when applied to new, unseen data. Furthermore, complex models with an extensive array of parameters are often difficult to implement in a clinical environment. While the collection of data such as age, ethnicity, family history of myopia and historical eye growth is relatively straightforward, assessing environmental risk factors, including outdoor time and near‐work habits, poses greater challenges.

The Predicting Myopia Onset and progression (PreMO) risk indicator addresses these challenges by synthesising readily accessible clinical data, including refractive and ocular biometric measurements, with the family history of myopia to provide an evidence‐based framework for eye care practitioners which provides guidance on the stratification and management of childhood myopia. The original PreMO risk indicator was presented as risk stratification tables available in a PDF format.^34^ It was specifically designed to stratify the risk of developing myopia and identify children to whom behavioural interventions and advice could be targeted. The PreMO risk indicator has now been developed into a web‐based app, which can be accessed free of cost by Optometrists and Ophthalmologists at myopiaonset.com.^35^ In this new web‐based format, the PreMO also functions as a communication aid, using colour‐coded infographics to support eye care practitioners in important conversations with at‐risk children and their parent/guardians.

For non‐myopic children, this myopia risk indicator stratifies the levels of risk based on four key factors: the child's age, parental myopia (whether neither, one or both parents are myopic), spherical equivalent refractive error (SER) of the least hyperopic/most emmetropic eye and axial length (AL). Table 1 summaries the risk score for children aged 6–8 (Table 1a) and 9–10 (Table 1b) years. Higher risk scores indicate a greater likelihood of developing myopia at an earlier age, categorised as follows: 0 = little/no risk, 1–3 = low risk, 4–6 = moderate risk and 7–9 = high risk (Table 2). If AL measures are not available, an estimation of risk associated with AL can be derived.^36^, ^37^ Predictive metrics were identified through risk analysis of the Northern Ireland Childhood Errors of Refraction (NICER) study, an extensive prospective UK population‐based investigation of white children.^38^, ^39^, ^40^, ^41^ The NICER study commenced in 2006 and recruited over 1000 white schoolchildren aged 6–7 and 12–13 years, establishing a cohort which was demographically representative of the Northern Irish population. A comprehensive assessment evaluating cycloplegic refraction, ocular biometry and visual status was conducted. Questionnaire data regarding family history of myopia, lifestyle and other environmental factors was also collected. Participating children underwent follow‐up examinations every 3 years across a span of 9 years. The evidence base which informs the PreMO risk indicator has been collated from a series of peer‐reviewed scientific publications, including those published by the NICER study group.^10^, ^36^, ^37^, ^38^, ^40^, ^42^, ^43^, ^44^, ^45^, ^46^ References and a detailed summary of this evidence are provided in Appendix S1.

**TABLE 1:** Risk factors used to calculate Predicting Myopia Onset and progression (PreMO) risk scores for (a) 6‐ to 8‐year‐old and (b) 9‐ to 10‐year‐old children. SER, spherical equivalent refraction.

Risk factor for myopia development	Score assigned
(a) *6‐ to 8‐year‐old children*	
Parental myopia	
Neither parent myopic	0
One parent myopic	2
Two parent myopic	3
Cycloplegic SER	
>+1.00 D	0
+0.75 to +1.00 D	2
<+0.75 D	3
Axial length	
<22.94 mm	0
22.94–23.11 mm	1
23.12–23.18 mm	2
≥23.19 mm	3
Risk score (0–9)	
(b) *9‐ to 10‐year‐old children*	
Parental myopia	
Neither parent myopic	0
One parent myopic	1
Two parent myopic	2
Cycloplegic SER	
>+0.875 D	0
+0.375 to +0.875 D	1
<+0.375 D	2
Axial length	
<23.33 mm	0
23.33–23.61 mm	1
≥23.62 mm	2
Risk score (0–6)	

**TABLE 2:** Predicted risk of developing myopia using the Predicting Myopia Onset and progression (PreMO) risk indicator framework.

PreMO risk score	Risk of myopia development	Predicted refractive outcome
0	Little/no risk	Likely to remain emmetropic
1–3	Low risk	Likely to be myopic by 16 years of age
4–6	Moderate risk	Likely to be myopic by 13 years of age
7–9	High risk	Likely to be myopic by 10 years of age

Despite the potential clinical implications of prediction models, a recent systematic review has highlighted a significant limitation: many existing myopia prediction models are based solely on data from school‐aged children and have not undergone comprehensive external validation.^47^ For predictive tools like the PreMO risk indicator to be considered reliable for widespread clinical use, it is important that they are validated using representative, independent datasets. Taking into account the known variability in the onset and progression rates of myopia across different ages and ethnicities,^24^, ^48^ the use of data sources which span a variety of age groups, ethnicities and geographical locations is essential for thorough validation.^47^ Such diversity is helpful to understand the predictive limits and accuracy of the model.

The aim of the present study was to evaluate the performance of the PreMO risk indicator when applied to independent data from a demographically varied, population‐based sample of UK children and a clinical sample of children living in Hong Kong (HK). The evaluation will shed light on the PreMO risk indicator's practicality and precision in assessing the risk of myopia among children who differ in ethnicity and geographical location.

## METHODS

This study involved the analysis of pre‐existing research and clinical databases. The performance of the PreMO risk indicator to predict future myopia development was evaluated using prospective data collected from independently sampled child populations in the UK and East Asia, specifically HK. Myopia was defined as SER ≤ −0.50 D, as per International Myopia Institute guidelines.^16^ To accurately reflect the application of the PreMO risk indicator in real‐world clinical practice, the evaluation did not exclude children with binocular vision anomalies or other ocular health issues from the analyses. This study involved the analysis of pre‐existing research and clinical databases.

### Data from the UK

Data collected as part of the Aston Eye Study (AES)—a large, multi‐ethnic population‐based study which commenced in 2006,^49^ were used to evaluate the performance of the PreMO risk indicator in UK children. Relevant clinical data, including age, AL, SER, parental history of myopia and ethnicity, were extracted from the AES data files. The subset of participants selected for this analysis was non‐myopic at age 6–7 years, as determined by cycloplegic autorefraction, with follow‐up refractive error data available at age 17–18 years. Detailed methodology pertaining to the AES has been published previously.^49^ Each participant's refractive ‘outcome’ at 17–18 years was used to determine the accuracy of the PreMO risk indicator in predicting future myopia. Given the extended 11‐year follow‐up interval of the AES, data were not available to evaluate the ability of the PreMO to predict the age of myopia onset (e.g., myopia onset before 10, 13 or 16 years of age), but rather whether myopia was present or absent by age 17–18 years.

### Data from HK

Clinical records of patients who attended the Optometry Clinic at the Hong Kong Polytechnic University (PolyU) between 2010 and 2021 were used to evaluate the PreMO risk indicator's performance in predicting future myopia in Asian children. The HK dataset was mined to identify children who were not myopic at the ages of (a) 6–8 years and/or (b) 9–10 years and had follow‐up data available up to the onset of myopia or until they reached at least 15 years of age. Children identified as non‐myopic at 6–8 years who remained non‐myopic at age 9–10 years were included in both analyses. It was assumed that children who developed myopia prior to turning 15 years old and had no further clinical records remained myopic, based on typical myopia progression patterns.^40^, ^50^ Over 97% of this clinical sample were of Chinese ethnicity.

The specifics of data collection procedures employed in the AES and PolyU Optometry Clinic are detailed in Appendix S2. All data were fully anonymised to ensure the identities of individual children could not be determined. The research protocol, which adhered strictly to the principles of the Declaration of Helsinki, was reviewed and approved by the Institutional Review Board at PolyU. The NICER and Aston Eye Studies received ethical approval from the Institutional Review Boards at Ulster and Aston Universities, respectively.

### Statistical analysis

All statistical analyses were performed using IBM SPSS Statistics software (Windows Version 28.0, ibm.com). Risk scores for future myopia were generated for non‐myopic participants within the 6‐ to 8‐ and 9‐ to 10‐year age cohorts as directed by the PreMO framework. These scores were computed based on the participant's cycloplegic SER, AL and parental myopia data (Table 1).

In the case of Hong Kong children, where data for both eyes were available, the SER and AL data from the least hyperopic or most emmetropic eye at the initial visit were used for analysis, following the guidelines of the PreMO framework.^43^ However, for the UK dataset, only mean SER and AL data—derived from both right and left eyes—were available and hence used in place of individual eye data.

The predictive accuracy of the PreMO risk indicator in forecasting future myopia was evaluated through Receiver Operator Characteristic (ROC) curve analysis. This method examined the sensitivity and specificity of risk scores generated at ages 6–8 or 9–10 years to predict the onset of myopia by the age of 15 years. The ROC curve visually depicts the distributions of risk for children who either developed ‘myopia’ (SER ≤ –0.50 D) or remained ‘non‐myopic’ (SER > –0.50 D) by age 15 years or older. The area under the curve (AUC) quantifies the discriminative ability of the PreMO risk indicator between these two outcomes, with a higher AUC indicating a better model in distinguishing between children who will or will not develop myopia. The Youden's *J* Index, defined as J=Sensitivity+Specificity−1, was used to identify the optimal risk score cut‐off to predict myopia onset. Additionally, Spearman's Rank Correlations were conducted to assess the relationship between the total PreMO risk score and the magnitude of SER at age 15 years or older.

To compare with previous research, sensitivity, specificity and AUC were also derived using singular predictors, that is, SER < +0.75 D^25^, ^40^ and AL ≥ 23.07 mm^40^, ^51^ at 6–8 years. AL ≥ 23.07 mm corresponds to the 75th centile of the NICER growth chart and was identified as a suitable cut‐off based on previous ROC curve analysis of NICER study data.

## RESULTS

Data from the AES and PolyU datasets identified three specific cohorts: 57 UK children aged 6–8 years (mean ± SD: 7.1 ± 0.35 years), 234 HK children aged 6–8 years (7.1 ± 0.76 years) and 75 HK children aged 9–10 years (9.7 ± 0.50 years). All participants were non‐myopic at baseline and follow‐up data were available to identify whether they were myopic or not at 15 years or older. Participant demographics are summarised in Table 3.

**TABLE 3:** Demographic details of each cohort (UK children aged 6–8 years, HK children aged 6–8 years and HK children aged 9–10 years) used to evaluate the predictive ability of the Predicting Myopia Onset and progression (PreMO) risk indicator for future myopia. HK, Hong Kong; SER, spherical equivalent refraction.

	Dataset	
Age group	UK	HK
*Age at baseline*		
6–8 years	Mean 7.1 ± 0.35 years	Mean 7.1 ± 0.76 years
9–10 years	–	Mean 9.7 ± 0.50 years
*Ethnicity*		
6–8 years	South Asian, *n* = 32	Western, *n* = 4
	White, *n* = 17	Chinese, *n* = 229
	Black, *n* = 7	Other, *n* = 1
	East Asian, *n* = 1	Western, *n* = 2
9–10 years	–	Chinese, *n* = 73
*Known refractive outcome at age ≥ 15* ^a^ *years*		
6–8 years	*n* = 57	*n* = 234
9–10 years	–	*n* = 75
*Known SER at age ≥ 15 years*		
6–8 years	*n* = 57	*n* = 26
9–10 years	–	*n* = 24

*Note*: Mean ± standard deviation.

^a^ The category ‘known refractive status at age ≥ 15 years’ includes children who developed myopia before age 15 (known refractive outcome) but with an unknown SER at age 15 or older. It was presumed that these children sustained their myopic condition after its onset. These children were included for all analyses except Spearman's Rank Correlations.

**FIGURE 1:**
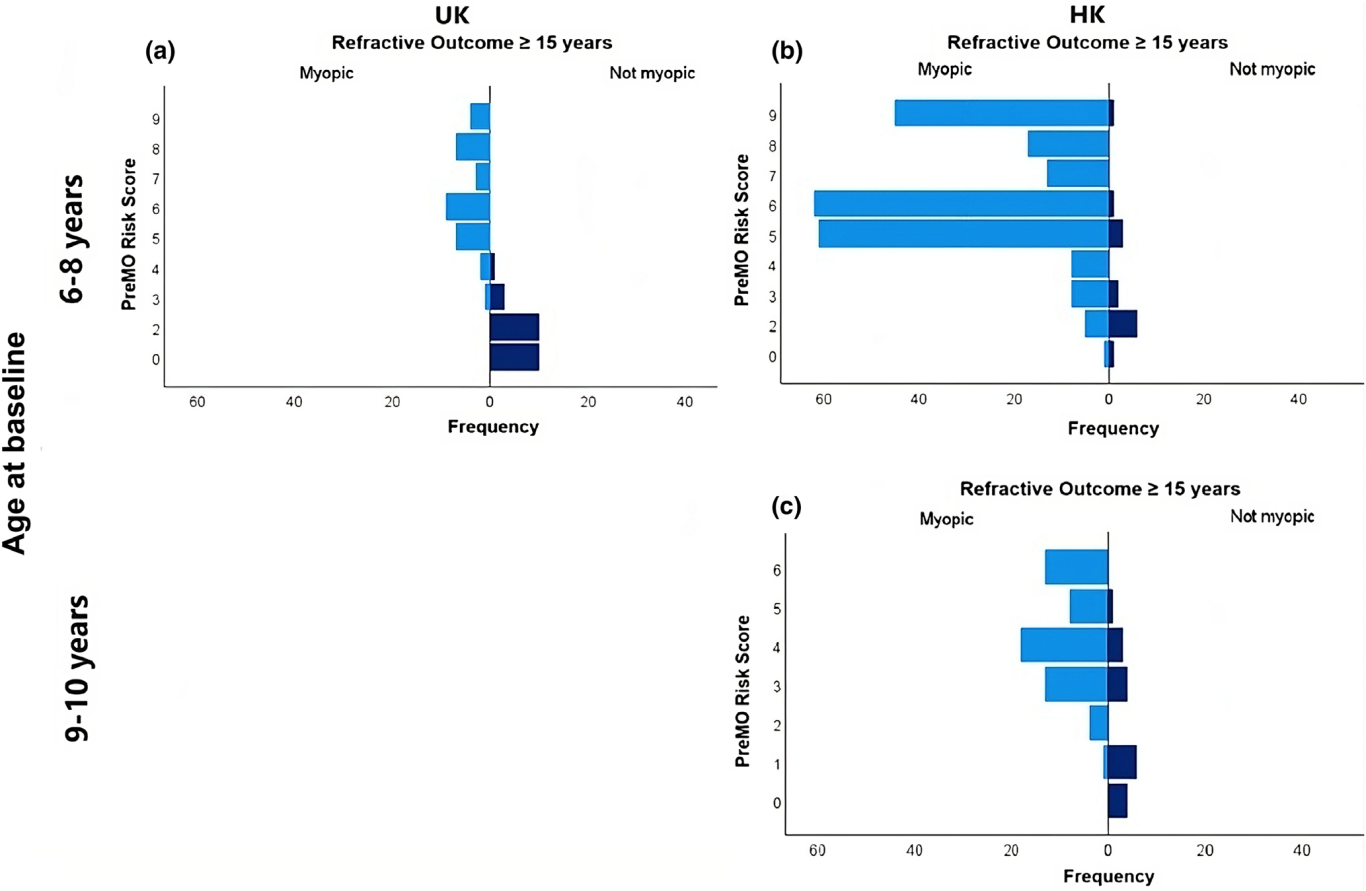
The distribution of Predicting Myopia Onset and progression (PreMO) risk indicator scores and subsequent refractive outcome at age of 15 years or older for three cohorts: (a) 6‐ to 8‐year‐old UK children, (b) 6‐ to 8‐year‐old HK children and (c) 9‐ to 10‐year‐old HK children. HK, Hong Kong.

Figure 1 illustrates the distribution of PreMO risk scores and refractive outcomes at age 15 or older in (a) 6‐ to 8‐year‐old UK children, (b) 6‐ to 8‐year‐old HK children and (c) 9‐ to 10‐year‐old HK children. Myopia was more prevalent in the HK cohort than in the UK cohort. Specifically, 94% (220 out of 234) of HK children, initially non‐myopic at the ages of 6–8 years, developed myopia by the age of 15 years or older. This incidence was approximately 1.6 times higher than that observed in UK children, where the incidence of myopia was 58% (33 out of 57).

The ROC curve analysis revealed excellent performance of the PreMO risk indicator in predicting future myopia development in both UK and HK children aged 6–8 years, with AUC of 0.926 and 0.834, respectively. This high predictive performance was also observed in HK children aged 9–10 years (AUC: 0.843). According to the highest Youden's *J* index, the optimal cut‐off value for distinguishing myopic from non‐myopic children was established as a risk score of ≥4. This optimum was consistent across all three cohorts. This risk score can therefore be considered the optimal threshold for predicting myopia development. Table 4 summarises the AUC, sensitivity, specificity and false positive rates in UK and HK datasets.

**TABLE 4:** Performance summary of Predicting Myopia Onset and progression (PreMO) risk indicator applied to the UK and HK datasets. AUC, area under the curve; CI, confidence interval; HK, Hong Kong.

Baseline age	Risk score cut‐off^a^	AUC (95% CI)	Sensitivity	Specificity	False positive rate
*UK*					
Age 6–8 years	≥4/9	0.996 (0.996–1.000)	0.97 (*n* = 32/33)	0.96 (*n* = 23/24)	0.04 (*n* = 1/24)
*HK*					
Age 6–8 years	≥4/9	0.834 (0.700–0.969)	0.94 (*n* = 206/220)	0. 64 (*n* = 9/14)	0.36 (*n* = 5/14)
Age 9–10 years	≥4/6	0.843 (0.725–0.961)	0.90 (*n* = 51/57)	0.72 (*n* = 13/18)	0.28 (*n* = 5/18)

^a^ Risk score cut‐offs were determined by choosing the highest Youden's *J* index (*J* = sensitivity + specificity − 1).

Leveraging ocular metrics (SER and AL) and parental history data collected at 6–8 years of age, the PreMO risk score cut‐off of ≥4 was highly sensitive in forecasting future myopia, achieving a sensitivity of 0.97 in UK children and 0.94 in HK children. This risk score demonstrated high specificity in UK children (specificity: 0.96) and lower specificity for HK children (specificity: 0.64). When applied to older HK children aged 9–10 years, the PreMO risk scores maintained high sensitivity (0.80) and specificity (0.72) for future myopia prediction. Corroborating these findings, Spearman's Rank Correlation revealed a high and statistically significant correlation between the pre‐myopic PreMO risk score and SER at the age of 15 years or older in both UK (ρ = −0.82, *p* < 0.001) and HK children (6–8 years: ρ = −0.58, *p* = 0.002; 9–10 years: ρ = −0.85, *p* < 0.001).

To compare with previous studies employing singular predictors,^25^, ^40^, ^51^ we evaluated the predictive performance using either baseline SER or AL for UK and HK children aged 6–8 years. When SER < +0.75 D served as the cut‐off in UK children, specificity was reduced compared to the PreMO risk indicator (Table 5). Application of SER as the sole predictor in HK children decreased the sensitivity (0.83) relative to the PreMO risk indicator, albeit with a higher specificity (0.86).

**TABLE 5:** Performance summary of singular predictors, spherical equivalent refraction (SER) < +0.75 D and axial length (AL) ≥ 23.07 mm, applied to UK and Hong Kong (HK) datasets at age 6–8 years.

Baseline age	Singular predictor							
	SER < +0.75 D				AL growth charts (≥75th centile 23.07 mm)			
	AUC (95% CI)	Sensitivity	Specificity	FPR	AUC (95% CI)	Sensitivity	Specificity	FPR
UK age 6–8 years								
	0.902 (0.806–0.997)	0.97 (*n* = 32/33)	0.83 (*n* = 20/24)	0.16 (*n* = 4/24)	0.758 (0.633–0.882)	0.52 (*n* = 17/33)	1.0 (*n* = 24/24)	0.0 (*n* = 0/24)
HK age 6–8 years								
	0.842 (0.732–0.952)	0.83 (*n* = 182/220)	0.86 (*n* = 12/14)	0.14 (*n* = 2/14)	0.594 (0.456–0.733)	0.33 (*n* = 73/220)	0.86 (*n* = 12/14)	0.14 (*n* = 2/14)

Abbreviations: AUC, area under the curve; CI, confidence interval; FPR, false positive rate.

Conversely, using the 75th centile on a European AL growth chart^40^ as the cut‐off resulted in suboptimal performance in predicting future myopia in children aged 6–8 years. Specifically, this approach exhibited poor sensitivity in both UK (0.52) and HK children (0.33).

## DISCUSSION

The PreMO risk indicator is designed to support clinicians and researchers wishing to identify children at risk of future myopia by utilising ocular biometric data (SER and AL) along with demographic information (age and parental myopia). By identifying pre‐myopic children and stratifying their risk of developing myopia in the future, this prediction and communication tool aids eye care practitioners in tailoring advice regarding lifestyle modifications and in implementing myopia management interventions aimed at mitigating the risk of myopia onset.

The application of the PreMO risk indicator to data from a multi‐ethnic UK cohort and a sample of children from HK showcased its high predictive accuracy for the development of myopia by the age of 15 years. The AUC was exceptionally high at 0.996 for the UK cohort and notable at 0.834 for the HK cohort. A PreMO risk score of 4 or higher was a strong indicator of future myopia in both populations, indicating high sensitivity across age groups and geographical locations.

However, as illustrated in Figure 1, PreMO risk scores proved more effective at distinguishing between children who did and did not develop myopia in the UK population than in HK. In the UK cohort, the likelihood of children with PreMO risk scores <4 developing myopia by the age of 15 years was remarkably low. In contrast, a noticeable portion of HK children with similarly low PreMO risk scores developed myopia. In other words, these results illustrate that HK children, including those without myopic parents or those with a hyperopic reserve, are still at risk of developing myopia. This disparity implies that environmental factors have a considerable influence on myopia development,^52^ especially within Asian populations like HK.^52^ The urban lifestyle experienced in many Asian cities often entails intense academic workloads and limited outdoor activities for children; environmental factors that have been established as contributors to the onset and progression of myopia.^12^, ^53^, ^54^, ^55^, ^56^, ^57^, ^58^

In HK children, comparable overall success in predicting future myopes was achieved using either the PreMO risk indicator (AUC 0.834) or SER cut‐offs by age (AUC 0.843 for SER < +0.75 D). Clinicians could apply either approach to support anti‐myopia behavioural and/or environmental modifications. However, when considering recently proposed prophylactic approaches to myopia management such as repeated low‐level red‐light therapy and low‐dose atropine, methods with higher specificity for predicting future myopia in the HK population may be more appropriate. Given that over 90% of children in the HK dataset developed myopia by the age of 15 years, it could be argued that identification of future myopes is of limited value in HK and similar populations, and that other functions of tools like the PreMO, such as communication and monitoring, may be more relevant.

A variety of myopia prediction models have been developed; their details extensively covered in recent literature and systematic reviews.^47^, ^59^ These range from relatively simple models which utilise baseline ocular biometric data^27^, ^28^, ^29^, ^60^, ^61^ to more complex frameworks, which integrate lifestyle and genetic information.^30^, ^33^, ^62^, ^63^, ^64^, ^65^ The models were designed to serve different purposes; some models use an age‐stratified approach to forecast the onset of myopia,^35^, ^66^, ^67^, ^68^ while others predict the likelihood of developing high myopia^29^, ^60^, ^69^, ^70^, ^71^ or estimate the expected rate of progression of myopia.^35^, ^48^, ^71^, ^72^, ^73^, ^74^ Other myopia risk ‘calculators’ include centile growth curves for AL and/or SER which can be used to predict the anticipated adult refractive error and/or AL.^40^, ^51^, ^72^, ^75^, ^76^ The advent of artificial intelligence has also created promising machine‐learning algorithms^29^, ^61^, ^63^, ^66^, ^71^, ^77^, ^78^, ^79^, ^80^; however, where the clarity in the reasoning process is essential for trust and adherence to ethical standards in healthcare settings, the opaque nature of these ‘black box’ models currently poses a challenge.

Comparing myopia prediction models which utilise a variety of predictors and definitions of myopia is inherently challenging. Because the PreMO risk indicator specifically targets the prediction of myopia onset, this discussion is confined to models which address the incidence of myopia. In this context, the AUC serves as a useful summary statistic to facilitate a fundamental comparison regarding the efficacy of these diverse models.

Concurring with the findings of the current study, age‐specific cycloplegic SER is recognised as the primary predictor for myopia development.^47^ The CLEERE study, which encompassed an ethnically diverse cohort, established age‐specific cut‐offs using cycloplegic SER as a singular predictor: <+0.75 D for 6‐year‐olds, <+0.50 D for 7‐ to 8‐year olds, <+0.25 D for 9‐ to 10‐year olds and <0 D for 11‐year olds.^25^ The NICER study also specified a SER cut‐off of ≤+0.63 D for European children aged 6–7 years.^40^ A cut‐off of ≤+0.50 D was identified in Chinese children aged 7–9 years in another investigation.^26^ These age‐specific cycloplegic SER criteria typically achieve high AUCs, ranging from 0.84 to 0.93.^25^, ^26^, ^32^, ^40^ Conversely, predictive models that depend solely on baseline AL are less effective, necessitating the integration of additional criteria to improve their accuracy. McCullough et al. and Ma et al. demonstrated that the AUC for predicting myopia incidence using AL alone dropped to 0.69 and 0.63, respectively; a marked decrease from the 0.87 and 0.86 AUCs obtained with cycloplegic SER.^26^, ^40^ Similarly, in the present study, cycloplegic SER as a singular indicator outperformed AL in predictive accuracy in both the UK and HK validation datasets (Table 5). Complex predictive models incorporating ocular biometric data with genetic and lifestyle factors, such as ethnicity, parental myopia and time spent outdoors and on near work, display a wide spectrum of performance, with AUCs or C‐statistics spanning from 0.68 to 0.98.^30^, ^32^, ^33^, ^63^, ^66^, ^67^, ^68^

The present study validated the PreMO risk indicator by using external datasets from the UK and HK, each with its distinct strengths and limitations. The UK dataset was sourced from the AES, a longitudinal, population‐based research study.^49^ Its primary strength lies in the application of standardised protocols that ensure data quality. However, the UK dataset was limited by its relatively small sample size, comprising only 57 children. A smaller sample size in some ethnic groups also limited the comparison of myopia predictive performance across ethnicities.

On the other hand, the HK dataset was obtained from the clinical records of the Optometry Clinic at the Hong Kong Polytechnic University. In contrast to the UK dataset, this clinical dataset might better represent the data typically encountered in real‐world clinical practice. Challenges associated with clinical datasets include non‐standardised protocols, data entry errors owing to the absence of routine quality control and incomplete data. Few children tested at the PolyU clinic who did not develop myopia by 15 years of age were retained in the clinical cohort until they reached 17–18 years. It is possible that some children included in the analysis as ‘non‐myopic’ at the age of 15 may have developed myopia in subsequent years. This limitation is not unique to the HK dataset, as it is also possible that some UK children recorded as non‐myopic at 17–18 years of age became myopic in adulthood. Potential bias may also arise if parents with myopia, being more aware of the risks, are more diligent in bringing their children for eye examinations and ensuring regular follow‐ups. This could potentially skew the data towards a higher likelihood of myopia development among these children. While this study aimed to validate the PreMO risk indicator in a real‐world clinical setting in HK, this potential bias should be considered when interpreting these findings.

The PreMO risk indictor can currently only predict risk of myopia onset for children 6 years or older, reflecting the age of the youngest participants in the NICER study. However, children may develop myopia before the age of 6 years, particularly in East‐Asian populations such as HK. Extending the PreMO's functionality to younger children would be a valuable direction for future research.

In conclusion, this study evaluated the efficacy of the PreMO risk indicator in forecasting whether non‐myopic children from the UK and HK would become myopic during childhood. The validation, encompassing diverse ages, ethnicities and geographical locations, adheres to the recommendations outlined by a recent systematic review.^47^ The PreMO risk indicator provides an evidence‐based framework incorporating age, SER, AL and parental myopia history with which clinicians can confidently forecast the likelihood of myopia development in young children. The low rate of false positives underscores the PreMO's utility in identifying UK children at risk of myopia, supporting eye care clinicians in efficiently targeting advice, follow‐up and prophylactic interventions. Its application in Asian populations, such as in HK, warrants consideration and clinical judgement, particularly if more ‘invasive’ measures, such as repeated low‐level red‐light therapy and low‐dose atropine, are being considered to delay or halt the progression of myopia. Future efforts will be directed towards refining and validating the indicator further, particularly with regard to strengthening its ability to support myopia management in a globally diverse paediatric population.

The present study forms the first validation exercise related to the PreMO risk indicator. Additional international collaborations and evaluations by those holding appropriate longitudinal data sets are welcomed to refine and enhance the tool further.

## AUTHOR CONTRIBUTIONS

**Jane M. Fulton:** Conceptualization (equal); data curation (equal); formal analysis (lead); methodology (equal); visualization (equal); writing – original draft (lead); writing – review and editing (equal). **Tsz Wing Leung:** Data curation (equal); investigation (equal); resources (equal); writing – original draft (equal); writing – review and editing (equal). **Sara J. McCullough:** Conceptualization (equal); methodology (equal); supervision (equal); visualization (equal); writing – review and editing (equal). **Kathryn J. Saunders:** Conceptualization (equal); methodology (equal); supervision (equal); visualization (equal); writing – review and editing (equal). **Nicola S. Logan:** Data curation (equal); investigation (equal); resources (equal); writing – review and editing (equal). **Carly S. Y. Lam:** Data curation (equal); investigation (equal); resources (equal); writing – review and editing (equal). **Lesley Doyle:** Conceptualization (lead); methodology (equal); supervision (equal); visualization (equal); writing – review and editing (equal).

## FUNDING INFORMATION

This work was supported by a Department for the Economy PhD Studentship (Northern Ireland), HK PolyU grant ZECH, the InnoHK initiative, the Innovation and Technology Fund for Better Living (ITB/FBL/8037/21/P) and the Hong Kong Special Administrative Region Government. The Northern Ireland Childhood Errors of Refraction Study and the Aston Eye Study were funded by the College of Optometrists, UK.

## CONFLICT OF INTEREST STATEMENT

SJM, KJS and LAD developed the PreMO risk indicator algorithms and risk stratification tables. The risk stratification tables have been publicly available online since 2020. With industry sponsorship, Wolffsohn Research Ltd converted PreMO into a browser‐based digital clinical tool, available free to users. PreMO is a not‐for‐profit enterprise. To‐date, the authors have received no financial support from Wolffsohn Research Ltd or their sponsors in relation to PreMO activity or usage. The authors have no other relevant conflicts of interest to declare.

## Supporting information


Data S1.
