# Supplementary material for: Cross‐population validation of the PreMO risk indicator for predicting myopia onset in children
Source: Ophthalmic Physiol Opt. 2024 Nov 18;45(1):89–99. doi: 10.1111/opo.13416 (PMC11629840; doi:10.1111/opo.13416)
Supplement: Supplementary file 1 — Data S1. [file OPO-45-89-s001.docx]

## Supplementary information

**Appendix 1**

**Evidence supporting the Predicting Myopia Onset and progression (PreMO) risk indicator**

Developed by researchers at Ulster University, the PreMO risk indicator is based on the findings of the Northern Ireland Childhood Errors of Refraction (NICER) study. This longitudinal research project has been pivotal in understanding the ocular changes that occur throughout childhood and adolescence. The evidence used to produce the PreMO risk indicator stems from a number of peer-reviewed scientific publications ^35–37,39,43,44^. The key evidence is summarised as follows:

**Risk factors for myopia development**

1. **Parental myopia**

- Children aged 6-7 years with at least one myopic parent were significantly more likely to become myopic by ages 10 or 13 than those with non-myopic parents, who typically developed myopia by 16 years^39^
- Children aged 6-7 years with a cycloplegic SER of ≤+0.19 D, at least one myopic parent, and axial length of ≥23.19 mm were the most susceptible to developing myopia by the age of 10 years^39^
- In white UK-based children, the presence of risk of one or two myopic parents increased the risk of developing myopia by 2.9 and 7.8 times, respectively, relative to children with no myopic parents^44^

1. **Refractive error**

- Earlier onset of myopia was correlated with higher final magnitudes of myopia^39^
- A cycloplegic SER of ≥+1.00 D at age 6-7 indicated a low likelihood of developing myopia^39^
- A cycloplegic SER of ≤+0.63 D at age 6-7 was the strongest predictor of myopia by age 9-10 (sensitivity: 90.91%, specificity: 76.53%)
- A cycloplegic SER of ≤+1.00 D at age 6-7 was the strongest predictor of myopia by age 12-13 (sensitivity: 90.91%, specificity: 44.33%)
- A cycloplegic SER of ≤+1.00D at age 6-7 was indicated increased likelihood of developing myopia by age 15-16 (sensitivity: 100.00%, specificity: 45.92%)
- A cycloplegic SER of ≤+0.25 D at age 9-10 was the strongest predictor of myopia by age 12-13 (sensitivity: 63.64%, specificity: 80.00%)
- A cycloplegic SER of ≤+0.875 D at age 9-10 was the strongest predictor for myopia by age 15-16 (sensitivity: 100.00%, specificity: 35.87%)

1. **Axial length**

- Children with an axial length of ≥22.94 mm at age 6-7 years were more likely to become myopic by age 15-16 (sensitivity: 71.43%, specificity: 71.43%)
- Children with an axial length of ≥23.12 mm at age 6-7 years were more likely to become myopic by age 12-13 (sensitivity: 54.55%, specificity: 79.80%)
- Children with an axial length of ≥23.19 mm at age 6-7 years were more likely to become myopic by age 9-10 (sensitivity: 54.55%, specificity: 82.65%)
- Children with an axial length of ≥23.62mm at age 9-10 years were more likely to become myopic by age 12-13 (sensitivity: 54.55%, specificity: 81.05%)
- Children with an axial length of ≥23.33 mm at age 9-10 years were more likely to become myopic by age 15-16 (sensitivity: 66.67%, specificity: 62.50%)

**Management of children at risk of myopia**

**Environmental factors**

- Implementation of environmental modifications and lifestyle advice for at-risk children may delay the onset of myopia and reduce the severity of myopia in later life^43^

**Age of onset**

- The likelihood of becoming myopic is threefold higher between the ages of 6-7 and 12-13 than between 12-13 and 18-19^37^
- Earlier onset leads to higher eventual magnitudes of myopia^39^

**Change in SER**

- A rapid annual change in cycloplegic SER (>0.25 D annually) can anticipate myopia onset within at least three years^39^

**Suggested recall for children at risk of myopia**

- Monitoring those children most at risk allows for early application of interventions when myopia manifests
- An average annual rate of change in cycloplegic SER of children in the NICER cohort who became myopic between ages 6-7 and 12-13 was –0.23D (IQR: –0.11 D to –0.45 D)^37^
- An average annual rate of change in cycloplegic SER of children and young adults in the NICER cohort who were myopic at 18-19 years was –0.10 D (IQR: –0.02 D to –0.17 D)^37^ between the ages of 12-13 and 18-19
- Given that an annual change in SER of –0.25 D is clinically significant, the NICER data suggest a recall interval of one year for children aged 6-7 to 12-13 years who are at risk of developing myopia and a two-year recall for those aged 6-7 to 12-13 years who are at low risk of myopia

**Appendix 2**

**Data collection procedures in the Aston Eye Study (AES) and Optometry Clinic at the Hong Kong Polytechnic University (PolyU)**

**AES**

Cycloplegia was achieved by instilling one drop of proxymetacaine hydrochloride 0.5% followed by one drop of cyclopentolate hydrochloride 1%. At least 20 minutes after instillation of the eye drops, distance autorefraction was carried out using the Shin-Nippon SRW-500019 binocular open-field autorefractor (rexxam.co.jp). The IOLMaster (zeiss.com/vision-care) was used to measure axial length. Family history of myopia was collected using a questionnaire^48^.

**PolyU**

One of three agents was instilled to induce cycloplegia: Mydrin-P (mixed eye drop containing 0.5% tropicamide + 0.5% phenylephrine), tropicamide 0.5%, or cyclopentolate hydrochloride 1%. Autorefraction was carried out using either Nidek ARK-510A (nidek-intl.com) or Topcon KR-800 (global.topcon.com), and axial length was measured using an IOLMaster (zeiss.com/vision-care) or AL-Scan (nidek-intl.com). Family history of myopia was determined by asking parents if they were myopic via telephone interview.
